# Treatment of adolescents with depression: the effect of transference interventions in a randomized controlled study of dynamic psychotherapy

**DOI:** 10.1186/1745-6215-13-159

**Published:** 2012-09-06

**Authors:** Randi Ulberg, Anne Grete Hersoug, Per Høglend

**Affiliations:** 1 Institute of Clinical Medicine, Department of Psychiatry, University of Oslo, Vinderen, PO Box 85 N-0319, Oslo, Norway; 2 Vestfold Hospital Trust, PO Box 2325, 3103, Tønsberg, Norway

**Keywords:** Psychodynamic, Transference, Randomized trial, Adolescent, Depression, Gender

## Abstract

**Background:**

Depression in adolescents seems to be a growing problem that causes mental suffering and prevents young people from joining the workforce. There is also a high risk of relapse during adult life. There is emerging evidence for the effect of psychodynamic psychotherapy in adolescents. In-session relational intervention (that is, transference intervention) is a key component of psychodynamic psychotherapy. However, whether depressed adolescents profit most from psychodynamic psychotherapy with or without transference interventions has not been stated.

**Object:**

The effect of transference interventions in depressed adolescents and the moderator moderating effect of quality of object relations, personality disorder and gender will be explored.

**Methods and study design:**

The First Experimental Study of Transference Work–In Teenagers (FEST–IT) will be a randomized clinical trial with a dismantling design. The study is aimed to explore the effects of transference work in psychodynamic psychotherapy for adolescents with depression. One hundred patients ages 16 to 18 years old will be randomized to one of two treatment groups, in both of which general psychodynamic techniques will be used. The patients will be treated over 28 weeks with either a moderate level of transference intervention or no transference intervention. Follow-up will be at 1 year after treatment termination. The outcome measures will be the Psychodynamic Functioning Scales (PFS), Inventory of Interpersonal Problems–Circumplex Version (IIP-C), Global Assessment of Functioning (GAF), and the total mean score of Symptom Checklist–90 (Global Severity Index; GSI), Beck Depression Inventory (BDI), and Montgomery Åsberg Rating Scale (MADRS). The quality of adolescents’ relationships will be a central focus of the study, and the Adolescent Relationship Scales (ARS) and Differentiation–Relatedness Scale (DRS) will also be used. Change will be assessed using linear-mixed models. Gender personality disorder (PD) and quality of object relations (QOR) will be the preselected putative moderators.

**Discussion:**

The object of this clinical trial is to explore the effect of transference interventions in psychodynamic psychotherapy in adolescents with a major depressive disorder. Using a randomized and dismantling design, we hope that the study will add more specific knowledge to the evidence base.

**Trial registration:**

ClinicalTrials.gov Identifier: NCT01531101

First Experimental Study of Transference work Work–In Teenagers (FEST-IT)

## Background

The number of young people (ages 16 to 24 years old) disabled by psychiatric disorders has tripled during the past 30 years. Among this group, 25% have been unable to work because of anxiety, depression, personality disorders or behavioral disorders. Young women especially have shown a significant increase in psychiatric symptoms, particularly depression and anxiety. Studies indicate that young women more than men of the same age and older women suffer from psychological distress and that girls present higher rates of depression than boys, whereas boys present higher rates of mania [1-4].

In addition to the mental suffering that these disorders cause, they carry a great risk of becoming chronic without adequate treatment, which may prevent individuals who have them from joining the workforce. The World Health Organization uses a measure, “years lost due to disability” (YLD; number of affected × seriousness of the disease × average duration of illness), to calculate the burden of different diseases on populations. Globally, depression has the highest YLD score [5,6].

Common treatment given to adults with depression is antidepressant medication or psychotherapy of short to intermediate duration. The effects of antidepressants might be questionable [7,8]. In the Treatment for Adolescents with Depression Study (TADS), adolescents with moderate to severe depression were observed to improve more with antidepressants and from a combination of antidepressants and cognitive-behavior therapy (CBT) than from CBT [9]. The effects of antidepressants on adolescents have not been explored to the same extent as they have for adults, and the results are contradictory. Antidepressant medication is recommended for adolescents in the USA [10], for example, but the UK NICE guidelines [11] do not advise antidepressant medication for a young person with moderate to severe depression, except in combination with a concurrent psychological therapy. According to a recent review, counseling and psychotherapy together is the treatment of choice in adolescents with major depressive disorder (MDD). However, in severe cases, antidepressants might be used for depression in adolescents in combination with psychotherapy [12].

In adolescent psychotherapy, as well as in adult psychotherapy, psychodynamic theory is one of the main theoretical approaches and is a treatment model based on principles described in the literature over several decades. Psychodynamics-based therapies comprise a wide spectrum of theoretical approaches; for example, mentalization, attachment, self-psychology object relations, and relational aspects [13-18]. *Transference* is a key concept in dynamic psychotherapy, and analysis of the transference distinguishes this treatment from other treatments. There is probably an array of active ingredients in the therapeutic action of psychotherapy with adolescents. Three of them might be analysis of transference, personality traits and gender.

*Analysis of transference* (transference work) is thought to be a fundamental technique in dynamic psychotherapy [19,20] with adults as well as with adolescents. The ongoing interaction between patient and psychotherapist is heavily influenced by the patient’s past relationships and affective experiences. A focus on the themes and conflicts that arise in the therapeutic relationship will therefore have immediate affective resonance and illuminate the nature of problems in the patient’s relationships outside therapy [21-24]. There are only two experimental and controlled studies in adults on the effect of transference work [25-27]. No experimental study has explored the effect of transference work in adolescents and identified for whom it works.

*Personality traits* influence the effect of psychotherapy [28]. However, the impact of difficulties in relational functioning and/or personality disorder on the effect of psychodynamic psychotherapy in adolescents has not been empirically explored [29].

*Gender* has not been found to be a general predictor in psychotherapy [28]. Few studies of adult patients [30-35] have explored gender as a moderator [36] of individual psychotherapy, which means testing whether female and male patients responded differently to different psychotherapy treatments. Ogrodniczuk *et al*. [31], Ulberg *et al*. [32,33], Frank *et al*. [34] and Barber *et al*. [35] found that gender moderates the effect of psychotherapy. The findings from research on the effect of gender in children and young people seem contradictory [37]. To the best of our knowledge, no experimental study has explored differences in response to transference interventions in dynamic psychotherapy between teenage girls and boys.

The field of psychotherapy research has made great advances in the past few decades. More than 1,500 studies have explored the effect of psychotherapy with children and adolescents [38]. However, randomized studies of the effect of dynamic psychotherapy in adolescents are few [39]. In a review of randomized controlled trials of CBT and psychodynamic psychotherapy, Thomas and colleagues did not find that studies differed significantly in quality [40]. However, one putative difference between CBT and psychodynamic psychotherapy might be that dynamic psychotherapy can have a long-term effect in adolescents. That is, the effect of treatment continues after termination in adults as well as in children and adolescents [41]. This is in contrast to some studies of CBT which did not give any evidence of a possible “sleeper effect” [42]. Meta-analytic reviews have provided support for the effectiveness of psychodynamic psychotherapy approaches to the treatment of mental disorders [43]. A strong evidence base for psychodynamic treatment of adolescents with depression is emerging [41,44].

Establishing that psychotherapy does have an effect does not, however, tell us much about *how, why* and *for whom* psychotherapy works. Therapists need to adjust and tailor their treatment approaches to the individual patient. Taking into account that individual psychotherapy is frequently the treatment of choice and is supposed to be beneficial for teenagers with depression, a research challenge is to further explore and establish empirically which techniques are critical to include for patients with different characteristics [45].

A randomized clinical trial (RCT) is a study in which an intervention is applied to diagnosed cases and analyzed against a comparison condition to determine the degree of change associated with treatment [46]. In a randomized controlled study, the included patients are randomized to a study treatment condition or to a different treatment condition or placebo. The response in a treatment group is compared to the response in a control condition. The efficacy of a treatment might thus be determined. Patients and evaluators should be blinded to treatment group. Efficacy studies emphasize the internal validity by (1) controlling the types of patients included in the study, (2) using manuals to standardize the treatment, (3) training the therapists prior to the study, (4) supervising the therapists during treatment, (5) monitoring the technique adherence and the “dose” of the therapy, (6) assigning participants randomly to treatments, and (7) selecting the primary outcome measures before the study begins [47].

Borkovec and Sibrava [48] stated that even the RCT method applied in psychotherapy research might ignore what specific therapist behavior causes the change in the patient during and after therapy. They maintained that psychotherapy studies mostly compare therapy modes that differ in a number of ways. Therefore, RCTs often do not generate significant basic knowledge.

Borkovec and Sibrava’s advice is to focus on identification of putative specific techniques that cause patient improvement. Studies should investigate treatment conditions that differ in only one component. Dismantling or component control designs contrast the complete therapy (for example, dynamic psychotherapy with transference interventions) with the same therapy with experimentally manipulated differences solely in one dimension (for example, dynamic psychotherapy without transference interventions). If the patient expectations and the quality of the patient–therapist relationship in both conditions are equal, therapist effects are minimized, and the quality with which interventions are given is equal in both groups, then the study offers the best possibility of detecting causal relationships between the specific technique and outcome.

There is a need for experimental studies. To provide this specific knowledge about adolescents, a RCT with a dismantling design will probably be the most suitable method. The present study is planned to be basic research on psychodynamic psychotherapy. With the intention being to contribute to the evidence base, the study will be an adjusted replication in adolescents with MDD, of the First Experimental Study of Transference–Interpretations. The Manual for Short-Term Psychoanalytic Therapy (STPP) from the Improving Mood with Psychoanalytic and Cognitive Therapies (IMPACT)–Study [49] and some of the measures in the Erica Process and Outcome Study (EPOS) [37] will be part of the assessment battery in FEST–IT.

### Related studies

#### First Experimental Study of Transference–Interpretations

The First Experimental Study of Transference–Interpretations (FEST) [27] is a RCT designed to explore specific long-term effects of transference work in time-limited dynamic psychotherapy in adults. Regular patients from general practice, private specialist practices and psychiatric outpatient departments were referred to the study therapists and assessed for eligibility. Inclusion criteria were liberal. One hundred patients were randomized to two different dynamic psychotherapies. Both treatments used general psychodynamic principles. One treatment avoided an interpretive focus on the ongoing patient–therapist interaction (comparison group). The other treatment used material from the patient–therapist interaction as the most important vehicle for clarifications, confrontations and interpretations (transference group).

In FEST, no significant differences between the two groups with regard to baseline characteristics could be detected. Treatment integrity was excellent [26]. Only use of transference work was significantly different between the two treatments. The two primary outcome measures were the Psychodynamic Functioning Scales (PFS) (clinician-rated) [25,26,50,51] and the Inventory of Interpersonal Problems (IIP–C) [52]. The two secondary outcome measures were the Global Assessment of Functioning Scale (GAS) and the Symptom Checklist-90-R (SCL-90) [53].

The Quality of Object Relation Scale (QOR), a measure of difficulties in relational functioning [54], was an important moderator of the effect of transference interventions [26,27]. Patients with the most problematic relationships with other people profited most from dynamic psychotherapy with transference interventions. Gender was also found to be a moderator [32,33]: Women experienced a significantly better treatment effect of transference work than men.

#### Improving Mood with Psychoanalytic and Cognitive Therapies study

The IMPACT study is an ongoing, large-scale RCT of depressed adolescents in the United Kingdom which will explore whether CBT or STPP is superior in reducing relapse compared with specialist clinical care in adolescents with unipolar depression. One hundred eighty patients will be randomized to each arm. The psychodynamic/psychoanalytic arm of the study is STPP. Patients will be assessed at pretreatment and at 6, 12, 36, 52 and 86 weeks after the beginning of therapy [49].

Erica Process and Outcome StudyEPOS [37] is a naturalistic, multicenter, pre-post study evaluating children’s functioning after psychotherapy. Quantitative and qualitative methods are used to describe factors underlying change.

## Methods/design

### Overview

The First Experimental Study on Transference Work–In Teenagers (FEST–IT) will be a RCT designed to explore effects of transference work in dynamic psychotherapy in adolescents with MDD. The design of the study is a so-called dismantling design in which a single component is added and/or varied to an existent treatment method (psychodynamic psychotherapy). Thus, the efficacy of a specific technique (transference interventions) can be identified.

### Aims

The primary hypothesis is that the transference group will have a more favorable course than the comparison group over time (that is, a significant improvement on the Psychodynamic Functioning Scales (PFS) and Inventory of Interpersonal Problems–Circumplex Version (IIP-C) and the measured depression by Mini International Neuropsychiatric Interview (M.I.N.I.), Beck Depression Inventory (BDI) and Montgomery Åsberg Rating Scale (MADRS) during the whole study period). The second hypothesis is that patients with a history of low QOR scale score and/or lacking personality disorders benefit more from therapy with transference work than from therapy without it. The third hypothesis is that female adolescents have better treatment effect of transference work than male adolescents do.

### Ethics

The Central Norway Regional Ethics Health Committee approved the study protocol (REK: 2011/1424 FEST-IT). The study is a replication of a study in adults in which patients in the two treatment groups responded, on average, equally well. The treatment will be manualized. The therapists will be experienced and specially trained, and the therapies will be supervised. Both of the two treatment modalities offered are frequently used, well-established psychotherapy methods. To study adolescent psychotherapy patients with self-rating instruments is a sensitive topic and requires a focus on the integrity of the individuals. No identifiable measures or parts of patient history will be kept outside the patient’s file. Only anonymous data will be kept in the database.

### Eligibility criteria

#### Inclusion criteria

Adolescents with a current *Diagnostic and Statistical Manual of Mental Disorders, Fourth Edition* (DSM-IV; American Psychiatric Association, 2000) unipolar MDD diagnosis will be included. Comorbidity is expected to be frequent. Written consent will be obtained from all patients.

#### Exclusion criteria

Patients with generalized learning difficulties, a pervasive developmental disorder, psychosis or substance abuse will be excluded [4,26]. To ensure that the sample is diverse and representative of the kind of patients referred to child and adolescent psychiatric outpatient departments and psychiatrists and psychologists in private practice in Norway, no other patients will be excluded.

### Patients

The patients will be recruited from among adolescents with symptoms of depression who are referred to private practice and child and adolescent outpatient departments in the South-Eastern Health Region, primarily in the Oslo area and in Vestfold County. The child and adolescent population in Vestfold County is 52,000, and that in the participating area of Oslo is 13,300. Oslo is an urban area. Vestfold County has rural and urban areas.

**Figure 1:**
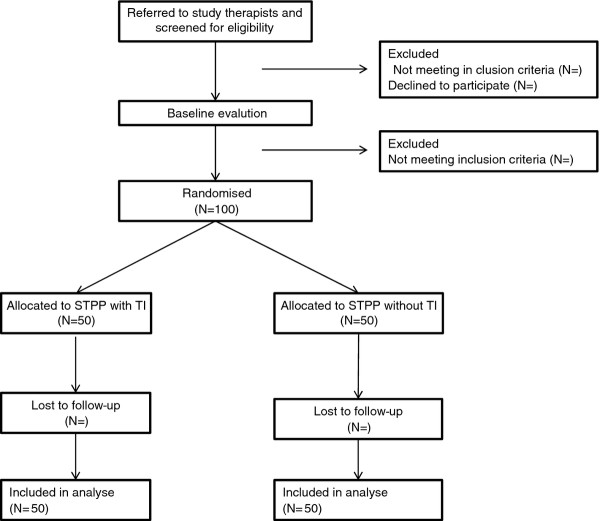
CONCORT flow diagram in a randomized controlled trial of dynamic psychotherapy with or without transference interventions (TIs).

One hundred participants will be recruited (Figure 1). The patients will be ages 16 through 18 years. At the first encounter, a referral form will be filled in [55]. The therapists will explore the patient’s current symptoms and situation, background, and developmental history. As screening tools, the patient will fill in the BDI, a self-report form [56] and the therapist will fill in the MADRS [57].

### Randomization procedures and methods used to minimize bias

#### Recruitment and baseline procedures

According to the ethical approval, the therapist and not one of the researchers will invite the patient to sign a consent form. If the patient scores above 10 on the BDI and/or above 15 on the MADRS, the therapists will inform the patient about the study and the patients will be asked to give written consent to participate. However, the patient will be advised to discuss this with someone they trust before agreeing to participate. The participants will then be contacted by an interviewer/evaluator, who will schedule an initial meeting. Thus, after the patients agree to participate, but before randomization, they will be diagnostically interviewed by an independent study interviewer using the M.I.N.I. 6.0.0 (Sheehan and Lecrubier, 2010), which is an interview tool used to diagnose psychiatric symptoms according to DSM-IV, and the Structured Interview for DSM-IV Personality (SIDP-IV) [58]. Before randomization each patient will also have a 1-hour Psychodynamic Interview (PI) modified after Malan [59] and Sifneos [60]. If the patient meets the criteria for major depression on M.I.N.I., the therapist (not the interviewer) will contact one of the researchers in the study and the patient will be randomized to one of two treatment groups.

Thus, after consent has been obtained and the baseline assessment has been carried out, a trial ID will be assigned and the randomization will be drawn by one of the main researchers in the study, who will not be the interviewer/evaluator. Only the researcher and the therapist will know the randomization arm. The same interviewer will assess the patient again at the two follow-up evaluations.

Each therapist will treat at least four patients with psychodynamic psychotherapy: two patients with and two patients without transference interventions. Thus, the randomization will be stratified. The order in which each therapist’s patients are allocated to each therapy mode will be drawn by one of the main researchers, a coordinator who will stay in direct contact with the therapist. To minimize bias that could arise from knowledge of treatment allocation, the following strategies will be employed: (1) The interviewers will be blinded to treatment allocation, (2) evaluators and therapists will not communicate with each other about the patient after inclusion, and (3) all evaluation and assessor interviews will be audiotaped and rerated by independent raters, who will be blinded to the randomization. If blindness is broken and the evaluator knows the randomization for one patient, all subsequent assessments will be carried out by an alternative evaluator [49]. Adherence to the randomization will be evaluated by blind raters by listening to three to five of the audio taped sessions from each patient and scoring them on the Specific Transference Technique Scales [27].

### Interventions: short-term psychodynamic/psychoanalytic psychotherapy (STPP) with transference interventions in comparison with STPP without transference interventions

#### Therapists

The patients will be treated by specially trained therapists. The therapists will be specialists in child and adolescent psychiatry, psychiatry or clinical psychology and will have at least 2 years of formal education (that is, practical and theoretical) in psychodynamic psychotherapy. They will be further trained in a 1-year program to provide dynamic psychotherapy with a moderate frequency of transference interventions (one to three per session) and dynamic psychotherapy without transference work. Peer supervision in groups will be offered to the therapists. This will help to maintain the quality of the therapies.

#### Treatment condition

Short-term psychodynamic/psychoanalytic psychotherapy (STPP) is a well-known treatment model [61,62]. The STPP manual from the IMPACT study [63] will be used as the manual for the treatment. The manual combines aspects of STPP that focus principally on techniques aimed at helping young people overcome developmental problems, as well as emphasizing the role of the interpretation of unconscious conflicts, attachment theory and the concepts of internal working models. With the agreement of the adolescent, parallel work with parents is included. Antidepressant medication may be added in severe cases according to the national guidelines in Norway [12]. FEST-IT will be an experimental study of the effect of dynamic psychotherapy with transference interventions on adolescents with depression [61,64]. The patients will be randomized to two treatment groups. The treatment in both groups will use general psychodynamic techniques [63]. The patients will be given 45-minute sessions. The treatment period will be 28 weeks. Therapies lasting less than 12 hours will be defined as dropouts. In the transference group, specific techniques will be prescribed, including the therapist (1) addresses transaction in the patient–therapist relationship, (2) encourages exploration of thoughts and feelings about the therapy and the therapist, and (3) interprets direct manifestations of transference and links repetitive interpersonal patterns to transactions between the patient and the therapist. In the comparison group, these techniques will be proscribed. In this group, the therapist will consistently use material about interpersonal relationships outside therapy as the basis for similar interventions (extratransference interventions). That means that in the transference group, analysis of the transference will be used with moderate intensity, whereas in the comparison group the therapists will avoid focusing on the patient–therapist relationship. Both treatments are mainly exploratory in nature. Patients in both treatment groups will be encouraged to explore sensitive topics. If the patient agrees, the parents and/or caregivers will be offered consultation by another therapist.

### Assessments and outcomes

#### Pretreatment: Interviews

Before randomization, each patient will be diagnosed according to DSM-IV criteria (M.I.N.I.6.0.0, SIDP–IV). See FEST-IT assessment battery (Table 1)Based on the information derived from an interview about present symptoms and situation, background and development history, as well as from information obtained from the Psychodynamic Interview (PI) (Sifneos [60] and Malan [59]), the patient’s Quality of Object Relations (QOR–2; relations to others and relation to family), the pretreatment scores on Global Assessment of Function Scale (GAF) over the past 2 weeks, and PFS [50] will be scored by two blinded raters. The Psychodynamic Functioning Scales (PFS) have the same format as GAF and measure psychological functioning. The six scales are as follows: Quality of Family Relationships, Quality of Friendships, Quality of Romantic/Sexual Relationships, Tolerance for Affects, Insight, and Problem Solving Capacity. Aspects of content validity, internal domain construct validity, interrater reliability, discriminant validity from symptom measures, and sensitivity for change in brief dynamic therapy have been established. In FEST–IT five of the six subscales will be used (quality of family relations, quality of friendships, tolerance for affects, insight, and problem-solving and adaptive capacity) [51,65,66]. The Differentiation-Relatedness Scale (DRS) [67] will also be scored by two blinded raters on the basis of the audiotaped interview.

**Table 1:** **Assessments administered at research baseline and each follow-up point throughout the trial**^**a**^

**Assessment points**	**Interviews**	**Measures**	**Self-report Questionnaires**	**Therapist questionnaires**
Baseline (0 weeks)	M.I.N.I., SIDP–IV, PI	QOR–2, PFS, GAF, DRS	BDI, PBI, Exp IIP–C, SCL–90, ARS,	MADRS
After session 3			WAI	WAI, FWC–24
After session 12			WAI, BDI, IIP–C, SCL–90, ARS	WAI, FWC–24, MADRS
After session 20			WAI, BDI	WAI, FWC–24, MADRS
Posttreatment	M.I.N.I., SIDP–IV, PI	PFS, GAF, DRS	WAI, BDI, IIP–C, SCL–90, ARS	WAI, FWC–24, MADRS
1-year follow-up	M.I.N.I., SIDP–IV, PI, MADRS	PFS, GAF, DRS	BDI, IIP–C, SCL–90, ARS	

^a^ARS, adolescent relationship scale; BDI, Beck Depression Inventory; DRS, Differentiation–Relatedness Scale; Exp, global expectancy; FWC–24, Feeling Word Checklist–24; GAF, Global Assessment of Functioning; IIP–C; Inventory of Interpersonal Problems–Circumplex Version; MADRS, Montgomery Åsberg Rating Scale; M.I.N.I., Mini-International Neuropsychiatric Interview; PBI, Parental Bonding Instrument; PI, psychodynamic interview; PFS, Psychodynamic Functioning Scale; QOR–2, Quality of Object Relation Score; SCL–90, Symptom Checklist–90; SIDP–IV, Structured Interview for DSM–IV Personality; WAI, Working Alliance Inventory.

#### Self-report

At pretreatment, the patient will complete the IIP-C [52], SCL-90 [53], and Parental Bonding Instrument Mother and Father (PBI) [68]. The patients will score the Adolescent Relationship Scale (ARS) which are visual analogue scales (VASs) used to evaluate the importance of friends, parents, siblings and quality of life [69], and the Global Expectancy (Exp), a visual analogue scale for patients [49] which indicates the confidence that the treatment will be helpful, will also be filled in.

#### During treatment

Working Alliance Inventory, the short form with 12 items (WAI) [70-72], will be filled in by the patient after sessions 3, 12, and 20. After session 12, the patients will also fill in the IIP–C, SCL–90-R, ARS and BDI. The WAI and Feeling Word Checklist–24 (FWC–24) [73] will be filled in by the therapist after sessions 3, 12 and 20. MADRS will be filled in after sessions 12 and 20.

#### Posttreatment

After session 28, the therapist will fill in the WAI, MADRS and Feeling Word Checklist–24. The patients will fill in the IIP–C, SCL–90-R, ARS, BDI and WAI. A blinded evaluator will interview the patient using the M.I.N.I. and SIDP–IV. PFS and GAF will be scored by two blinded raters on the basis of an audiotaped interview. The DRS will be scored.

### Follow-up assessment

#### 1-year follow-up

The patients will fill in the IIP–C, SCL–90-R, ARS and BDI. A blinded evaluator will interview the patient using the M.I.N.I. and SIDP–IV. The PFS and GAF will be scored by two blinded raters on the basis of an audiotaped interview. The DRS will be scored.

### Planned investigations

FEST–IT will be a randomized trial that compares short-term psychodynamic psychotherapy with and without transference interpretation to reduce relapse in adolescents with DSM-IV-defined MDD. The patients will be randomly allocated to STPP with or without transference interventions. This is a dismantling design in which a single component is added and/or varied to an existing treatment approach. The therapy is manualized. Outcomes will be assessed at planned follow-up points by outcome evaluators unaware of the treatment allocation. The therapists will be psychodynamically trained therapists who will undergo a 1-year training program succeeded by peer supervision in groups. This will help maintain the quality of the therapies. The therapies will be audiotaped to evaluate treatment adherence.

### Sample size and power

On the basis of standard posttest comparison of the two groups, 100 patients in this study will be enough to reveal a moderate effect size (0.55), with a significance level of 0.05 and a power of 0.80 [74]. In this study, an effect size (ES) of 0.55 equals 3.3 steps on the GAF scale, since the standard deviation across groups at posttreatment is 6. By using a pre-post treatment design, the power will increase. Adding data from 1-year follow-up will increase the power to detect differences even more. An α level of 0.10 has been decided on *a priori* for the moderator analyses and the subgroup analyses in this study to offset the risk of type II errors [74].

### Statistical analysis plan

Intention-to-treat analysis will be performed. Characteristics of the treatment groups will be described at baseline. A linear mixed model will be used to analyze treatment effects of transference interventions in depressed adolescents. The statistical analyses will be performed with the linear mixed model using all data from all patients during the whole study period. Treatment response in FEST–IT will be the effect of transference intervention in depressed adolescents. The primary hypothesis will be explored by using this equation [75]:

Outcome measure = intercept + time + time × treatment + QOR + time × treatment × QOR + residuals.

Both predictors and moderators are pretreatment variables that might affect the strength or direction of the treatment response. Predictors do so regardless of treatment condition. Moderators [76] differentially influence outcome, however, depending on treatment condition [77,78]. Moderators specified in advance are QOR, Personality Disorder, and gender. Those patient variables will be explored as predictors or moderators of outcome.

To explore the effects of moderators in FEST–IT, the following model will be used [75]:

Outcome measure = intercept + time + time × treatment + QOR + time × treatment × QOR + residuals.

### Economic evaluation

Patients referred to private practice and child and adolescent outpatient departments in the South-Eastern Health Region, Norway, will be treated, and the treatment will be evaluated using the FEST–IT. Current resources do not allow for including cost-effectiveness analyses of the two treatments. However, we are aiming at providing funding for such analyses during the process of the study.

## Discussion

Depression in adolescents might relapse, and treating and preventing further symptoms can help young people find their way into adult relationships and into the workforce. To guide therapists in which therapy works for patients with different characteristics, there is a need for more studies to investigate the effect of psychotherapy.

According to Borkovec and Costello [79], the study will have a RCT dismantling design. Differences in psychodynamic psychotherapy with and without transference interventions will be explored. Thus solely one technique, the transference interventions, will be experimentally manipulated. The present design will fulfill RCT requirements: Treatment will be based on clear descriptions from a manual. Power calculation will be made. Primary and secondary hypotheses will be defined. The patients will be unaware of which treatment group they have been assigned to. Real patients will be randomized to therapy (treatment and comparison group) and treated by specially trained therapists. The patients will be diagnosed on the basis of formal interviews (M.I.N.I. and SIDP-IV). Assessments will be conducted at three time points with the primary outcome variables using the PFS-5 and the secondary outcome measure using GAF. Assessments will also be undertaken at four time points using the primary outcome measure IIP–C and the secondary outcome measure GSI. Evaluators will be blinded into which treatment group patients were placed. Reliability will be tested between the raters on the GAF, PFS–5 and QOR − 2. Moderators specified in advance (QOR–2, PD and gender). The present study aims to explore the effect of transference interventions in psychodynamic psychotherapy, and we hope it will add more knowledge to the evidence base for treatment of adolescents with major depression.

### Trial status

This study is an ongoing study that is not completed.

## Abbreviations

ARS: adolescent relationship scale; BDI: Beck Depression Inventory; CBT: cognitive-behavior therapy; DSM-IV: Diagnostic and Statistical Manual of Mental Disorders, Fourth Edition; EPOS: Erica Process and Outcome Study; Exp: global expectancy; FEST: First Experimental Study Of Transference–Interpretations; FEST-IT: First Experimental Study of Transference Work–In Teenagers; FWC-24: Feeling Word Checklist–24; GAF: Global Assessment of Functioning; GSI: Global Severity Index (Total Mean Score of Symptom Checklist–90); IIP-C: Inventory of Interpersonal Problems–Circumplex Version; IMPACT: Improving Mood with Psychoanalytic and Cognitive Therapies; MADRS: Montgomery Åsberg Rating Scale; M.I.N.I.6.0.0, Mini-International Neuropsychiatric Interview; MDD: major depressive disorder; PBI: Parental Bonding Instrument; PI: psychodynamic interview; PFS: Psychodynamic Functioning Scale; QOR–2: Quality of Object Relation Score; RCT: randomized clinical trial; SIDP-IV: Structured Interview for DSM-IV Personality; STPP: short-term psychodynamic/psychoanalytic therapy; WAI: Working Alliance Inventory.

## Competing interests

The authors declare that they have no competing interests.

## Authors’ contributions

All authors contributed to the design of the FEST–IT trial protocol, to discussions regarding alternative designs and to writing all parts of the paper. All authors read and approved the final manuscript.
